# Prognostic value of microRNAs in heart failure: A meta-analysis

**DOI:** 10.1097/MD.0000000000027744

**Published:** 2021-11-19

**Authors:** Jie Yang, Xue-Song Yang, Shao-Wei Fan, Xiao-Yu Zhao, Chao Li, Zheng-Yao Zhao, Hui-Juan Pei, Lu Qiu, Xin Zhuang, Chuan-Hua Yang

**Affiliations:** a Department of Cardiovascular, Affiliated Hospital of Shandong University of Traditional Chinese Medicine, No. 16369, Jingshi Road, Jinan, China.; b Department of Vascular Surgery, Affiliated Hospital of Shandong University of Traditional Chinese Medicine, Jinan, China; c College of Traditional Chinese Medicine, Shandong University of Traditional Chinese Medicine, Jinan, China; d Department of Cardiovascular, Affiliated Hospital of Shandong University of Traditional Chinese Medicine, No. 16369, Jingshi Road, Jinan, China.

**Keywords:** circulating microRNAs, heart failure, meta-analysis, prognosis

## Abstract

**Background:**

Reported studies have shown that expression levels of microRNAs (miRNAs) are related to survival time of patients with heart failure (HF). A systematic review and meta-analysis were conducted to study circulating miRNAs expression and patient outcome.

**Methods:**

Meta-analysis estimating expression levels of circulating miRNAs in HF patients from January 2010 until June 30, 2018, through conducting online searches in Pub Med, Cochrane Database of Systematic, EMBASE and Web of Science and reviewed by 2 independent researchers. Using pooled hazard ratio with a 95% confidence interval to assess the correlation between miRNAs expression levels and overall survival.

**Results:**

Four relevant articles assessing 19 circulating miRNAs in 867 patients were included. In conclusion, the meta-analysis results suggest that HF patients with low expression of serum *miR-1*, *miR-423-5p*, *miR-126*, *miR-21*, *miR-23*, *miR-30d*, *miR-18a-5p*, *miR-16-5p*, *miR-18b-5p*, *miR-27a-3p*, *miR-26b-5p*, *miR-30e-5p*, *miR-106a-5p*, *miR-233-3P*, *miR-301a-3p*, *miR-423-3P,* and *miR-128* have significantly worse overall survival (*P* *<* *.05*). Among them, *miR-18a-5p, miR-18b-5p, miR-30d*, *miR-30e-5p*, and *miR-423-5p* are strong biomarkers of prognosis in HF.

## 1 Introduction

In recent years, circulating microRNAs have received increasing attention in the study of cardiovascular disease, and although they have not yet reached the clinical application stage, they have long been shown to play an important role in most cardiovascular diseases. Accumulating studies recognize that circulating microRNAs (miRNAs) are involved in the molecular mechanisms involved in the pathophysiological processes of coronary atherosclerotic plaques and therefore have potential diagnostic and therapeutic value.^[1]^ For example, miR-17-5p, miR-33a, miR-221, miR-222 were mentioned in 1 paper as useful biomarkers for the diagnosis of atherosclerosis, while miR-200c is a biomarker of atherosclerotic plaque progression and can be used to identify patients at high clinical risk of sclerosis.^[2]^ One study found that circulating miR-122 levels were higher in patients with coronary heart disease (CHD) than in controls, and the authors suggest that serum levels of miR-122 may also be used to differentiate the severity of coronary atherosclerotic lesions.^[3]^ In addition, serum miR-9-3p and miR-144-3p are recognized as the most important functional miRNAs and are the most important signaling pathways in dilated cardiomyopathy (DCM). Huang et al^[4]^ researched that circulating microRNAs such as miR-9-3p, miR-21-3p, miR-144-3p, and miR-144-5p play a key role in the development and pathogenesis of DCM. Similarly, many miRNAs have been found to be associated with heart failure.

Heart failure (HF) is a complex clinical syndrome caused by cardiac structural and functional disorders, mainly including dyspnea, fatigue, poor exercise tolerance, and fluid retention.^[5–7]^ Heart failure is the final stage in the progression of various cardiovascular diseases, which will greatly reduce the quality of life of patients with it and even endanger their lives.

The morbidity and mortality of heart failure remain high in the world, and heart failure affects nearly 8.823 billion people worldwide, causing 270,000 deaths each year.^[8]^ Gender, age, ethnic, complications, and environment are all influencing factors. According to the US National Health and Nutrition Examination Survey, 5.7 million Americans suffer from heart failure, and that number is likely to rise to at least 8 million by 2030.^[9]^ Data show that the prevalence rate of heart failure in developed countries is 1.5% to 2.0%, and that of people over 70 years old is more than 10%. In China, the prevalence rate of heart failure among adults aged 35 to 74 is 0.9%.^[10]^

Heart failure is a widespread and devastating cardiovascular disease, so it is of great significance to improve the prediction accuracy of heart failure, provide treatment as soon as possible and improve the quality of life of patients with heart failure.

It is reported that expression levels of miRNAs in patients with heart failure are closely related to survival time.^[11]^ The purpose of this study is to assess the prognostic value of circulating miRNAs in HF patients.

### 1.1 MicroRNAs

The mature miRNAs are short single-stranded endogenous non-coding RNAs (19-23 base pairs in length),^[11]^ which can regulate gene expression after transcription and are widely distributed in plants and animals.^[12–14]^ The formation process of miRNAs is complex. The initial miRNA is transcribed by RNA Polymerase II in the inner rotor or intergenic region and is 1 to 3 kb in length, and is then developed into pre-miRNAs (stem-structured precursor miRNAs) in the nucleus, under the influence of both Drosha (RNase III) and Pasha (double-stranded RNA-binding protein). The pre-miRNAs are 70 to 100 nucleotides in length.^[15–18]^

Exportin-5 mediates the transport of pre-miRNAs from the nucleus into the cytoplasm, which are then cleaved into a double-stranded oligonucleotide (mature double-stranded miRNA) (18-24 nucleotides in length) by the RNase III Dicer.^[19,20]^ miRNAs are important for a number of biological processes, including the regulation of gene expression, and have been implicated in the pathogenesis of various disorders, including cardiovascular diseases.^[21,22]^

### 1.2 Heart remodeling and heart failure

Heart failure is typically associated with cardiac remodeling.^[23]^ Cardiac remodeling is a crucial mechanism in HF progression, and is an important factor in cardiac function and prognosis of heart failure.^[24]^ The 3 main features of cardiac remodeling include pathological cardiomyocyte hypertrophy, excessive cardiac extracellular matrix fibrosis, and cardiomyocyte apoptosis.^[25]^ Normal cardiomyocytes are surrounded by collagen fibers and fibroblast which secrete extracellular matrix proteins to cope with pathological stress or myocardial infarction. However, excessive secretion of extracellular matrix proteins leads to myocardial fibrosis, causing mechanical stiffness and contractile dysfunction. Apoptosis of cardiomyocytes can occur through a variety of pathways, accelerating the progression of heart failure.

In addition, studies have shown that heart failure is a prethrombotic state, suggesting that thrombosis and embolism can lead to heart failure events and reduced ejection fraction.^[26]^ It has been reported that heart failure is characterized by autonomic nervous imbalance, sympathetic hyperactivity and parasympathetic dystonia. Oxidative stress is thought to lead to myocardial injury and inflammation, leading to the progression of heart failure.^[27]^

### 1.3 Heart failure-related miRNAs

Since the correlation of miRNAs with heart failure was first reported in 2006,^[28]^ numerous miRNAs have been shown to be associated with the development of heart failure, including, miRNA-21, miRNA-1, miRNA-208, miRNA-499, and miRNA-133, among others.^[29,30]^

## 2 Methods

All analyses were based on previous published studies, thus no ethical approval and patient consent are required.

### 2.1 Literature inclusion and exclusion criteria

Eligibility criteria. A randomized controlled trial was conducted using the blinded method or distributed concealment method. Literatures included in English were included only. The study targets were patients diagnosed with heart failure in accordance with the *2016 ESC Guidelines for the diagnosis and treatment of acute and chronic heart failure*.^[31]^ The target population was not limited in race, age or gender. The date of publication was limited from January 1, 2010 to June 30, 2018. The data content of the literature includes data analysis tables, hazard ratio (HR), and the corresponding 95% confidence interval (CI), except for the survival curve. The outcome index is the result of the original papers.

Exclusion criteria. Literature from which relevant data could not be extracted or for which full-text articles were not obtained were excluded. Reviews and case reports were all excluded, as were papers that did not meet the inclusion criteria.

### 2.2 Search strategy

A systematic search in PubMed, EMBASE, Science Web, and Cochrane Database was conducted incorporating literature published from January 2010 to June 2018. The English titles or keywords were heart failure or MiRNA or MicroRNA.

### 2.3 Literature screening

Documents were screened by 2 researchers independently by reading the title and abstract, followed by the full text if the paper met the inclusion criteria. Only papers that were assigned by both researchers to be included were taken into account. A third researcher would include or exclude papers in the event of discrepancy between the 2 researchers.

### 2.4 Literature quality evaluation

The 2 researchers assessed the quality of the included studies independently based on the bias risk assessment tool of randomized controlled trials recommended in the Cochrane System Evaluator's Handbook 5.1.0. In the event of discrepancy, the inclusion or elimination was determined jointly by the 2 researchers or a third researcher decided whether to include or exclude the paper.

### 2.5 Statistical processing

Data processing and analysis were performed using R 3.6.0 statistical software (GNU system). The HR was used as the effect index, and both the point estimate and the 95% CI of each effect quantity are given. The *I*^2^ value (test level α = 0.05) was calculated using the Cochrane Q test. Intergroup heterogeneity analysis was performed for each trial using *I*^2^ to determine the heterogeneity. If there was no heterogeneity between the studies (*P* ≥. 05 and *I*^2^ ≤ 50%), the fixed effect model would be used for analysis, otherwise, the random effect model would be used for analysis and the reasons for heterogeneity would be discussed.

## 3 Results

### 3.1 Literature search results and basic information of included studies

The research formula retrieved a total of 4, 490 related articles, including 1930 articles from PubMed, 0 articles from EMBASE, 0 articles from Cochrane Database of Systematic Reviews, and 2560 articles searched through other methods. Two thousand three hundred fifty-one articles remained after removing duplications and 1390 articles were retained after the initial tile and abstract screening. After reading the full text of 1390 articles, 1030 articles were excluded. A further review of the remaining literature shows that, compared with the latest literature, the 4 articles published from 2015 to 2017 are more suitable for this study. Finally, 4 randomized controlled trials were included,^[32–35]^ with 867 patients involved, including 695 patients in the experimental group and 172 patients in the control group. Nineteen miRNAs in serum from the 4 relevant articles were ultimately assessed.

**Figure 1:**
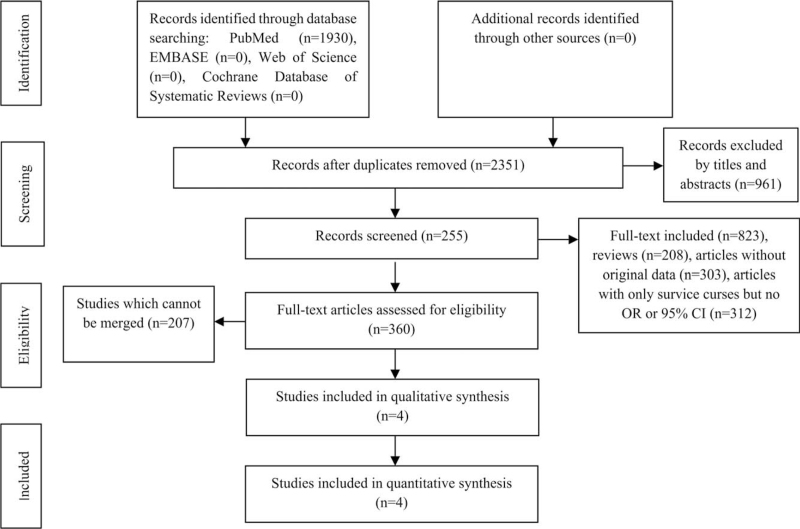
Flow chart detailing the literature search and selection.

Figure 1 shows a flow chart of the detailed selection process for this study. The basic information of the literature included in the study is shown in Table 1. Table 2 is a summary of all relevant microRNAs in the retrieved literature; Table 3 is the repetitive microRNAs in Table 2. There are 3 repetitive microRNAs: *miR-423-5p, miR-30, and miR-18*. As shown in Table 4, low expression of circulating *miR-423-5p* (HR = 0.66, 95% CI = 0.53-0.84), *miR-30* (HR = 0.63, 95% CI = 0.46-0.85), and *miR-18* (HR = 0.59, 95% CI = 0.43-0.80) are associated with worse overall survival of heart failure patients. The results of the subgroup analysis (Country, Material, and miRNA) are shown in Table 5.

**Table 1:** This Table is the collation of the information of the articles included in the article.

No.	Research topic	Author	Country	Material	Disease
1	Circulating microRNAs and Outcome in Patients with Acute Heart Failure ^[28]^	Seronde MF, Vausort M, Gayat E, et al^[28]^	France	Plasma	Acute Heart Failure
2	The prognostic value of circulating microRNAs in heart failure: preliminary results from a genome-wide expression study^[29]^	Cakmaka HA, Coskunpinarb E, Ikitimurc B, et al^[29]^	Turkey	Serum	Chronic Congestive Heart Failure
3	Circulating miR-30d Predicts Survival in Patients with Acute Heart Failure ^[30]^	Xiao JJ, Gao RR, Bei YH, et al^[30]^	China	Serum	Acute Heart Failure
4	Signature of circulating microRNAs in patients with acute heart failure ^[31]^	Ekaterina S. Ovchinnikova, Daniela Schmitter, Eline L. Vegter, et al^[31]^	Netherlands	Plasma	Acute Heart Failure

**Table 2:** Summary of miRNA profiles involved in all of the studies, including the HR value and confidence interval of miRNA.

Study	MicroRNA	HR	Ll	Ul
1	miR-423-5p	0.70	0.53	0.93
1	miR-126	0.96	0.92	1.01
1	miR-23	0.95	0.91	1.00
1	miR-21	0.99	0.98	1.00
1	miR-1	1.00	0.99	1.00
3	miR-30d	0.61	0.41	0.911
4	let-7i-5p	0.51	0.31	0.84
4	miR-16-5p	0.67	0.42	1.07
4	miR-18a-5p	0.62	0.42	0.91
4	miR-18b-5p	0.54	0.33	0.88
4	miR-26b-5p	0.92	0.60	1.40
4	miR-27a-3p	0.73	0.48	1.09
4	miR-30e-5p	0.65	0.41	1.02
4	miR-106a-5p	0.67	0.44	1.01
4	miR-128	0.80	0.51	1.25
4	miR-199a-3p	0.79	0.52	1.20
4	miR-233-3p	0.64	0.43	0.97
4	miR-301a-3p	0.56	0.38	0.84
4	miR-423-3p	0.74	0.48	1.14
4	miR-423-5p	0.59	0.38	0.92
4	miR-652-3p	0.65	0.42	1.00

HR = hazard ratios of microRNA, LI = the lower limit of 95% confidence interval, Study = according to the number of Table 1 to reduce the length of the table, UI = the upper limit of 95% confidence interval.

**Table 3:** Summary of miRNA profiles involved in the studies involved, including the HR value and confidence interval of miRNA.

Study	MicroRNA	HR	Ll	Ul
1	miR-423-5p	0.70	0.53	0.93
4	miR-423-5p	0.59	0.38	0.92
3	miR-30d	0.61	0.41	0.91
4	miR-30e-5p	0.65	0.41	1.03
4	miR-18a-5p	0.62	0.42	0.91
4	miR-18b-5p	0.54	0.33	0.87

HR = hazard ratios of microRNA, LI = the lower limit of 95% confidence interval, Study = according to the number of Table 1 to reduce the length of the table, UI = the upper limit of 95% confidence interval.

**Table 4:** The relationship between miRNA expression and clinical outcome.

Outcome	Study	MicroRNA	HR	Ll	Ul
Low expression-worse OS	1	miR-423-5p	0.7	0.53	0.93
	4	miR-423-5p	0.59	0.38	0.92
	3	miR-30d	0.61	0.41	0.91
	4	miR-30e-5p	0.65	0.41	1.03
	4	miR-18a-5p	0.62	0.42	0.91
	4	miR-18b-5p	0.54	0.33	0.87

HR = hazard ratios of microRNA, LI = the lower limit of 95% confidence interval, OS = overall survival, Study = according to the number of Table 1 to reduce the length of the table, UI = the upper limit of 95% confidence interval.

**Table 5:** The results of subgroup analysis are as follows.

Subgroup	No. of studies	HR (95% CI)	*I*^2^(%)	*P*
Country				
France	1	0.70 (0.53, 0.92)		
China	1	0.61 (0.41, 0.91)		
Netherlands	4	0.60 (0.48, 0.75)	0%	.95
Material				
Plasma	5	0.64 (0.54, 0.76)	0%	.90
Serum	1	0.61 (0.41, 0.91)		
MicroRNA				
miR-423-5p	2	0.66 (0.53, 0.84)	0%	.51
miR-30	2	0.63 (0.46, 0.85)	0%	.85
miR-18	2	0.59 (0.43, 0.80)	0%	.66

CI = confidence interval, HR = hazard ratios of microRNA, *I*^2^ = the heterogeneity between the various studies, No. of studies = number of studies included, *P* = *P* value.

### 3.2 Methodological quality evaluation results

The 4 papers included all mentioned randomization, but the specific random methods were mentioned in none of them, nor were they clearly stated.

### 3.3 Meta-analysis results

**Figure 2:**
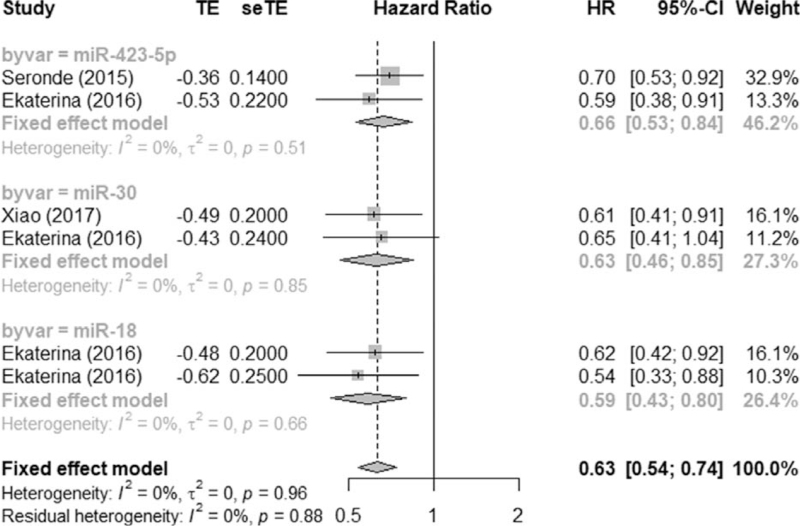
Forest diagram showing the results of effect integration of the HR values in the included literature through a fixed effects model.

**Figure 3:**
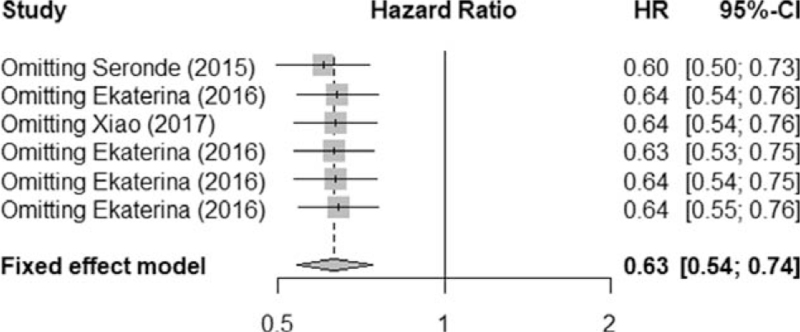
The sensitivity analysis is shown in the figure.

All 4 studies reported different miRNAs in heart failure. The effect integration of all the literature's HR indicators found that *I*^2^ = 0% < 50%, indicating that the included literature is not heterogeneous (heterogeneity test χ^2^ = 0, *P* = .96) and requires integration through a fixed effects model, see Figures 2 and 3 for details. Meta-analysis showed that the integrated HR is 0.63 (95% CI, 0.54-0.74).

### 3.4 Sensitivity analysis

A sensitivity analysis is an important part of a meta-analysis as it aims to determine the robustness of the observed outcomes. If results remain consistent across the sensitivity analysis, the results can be considered robust. A meta-analysis of the remaining research was performed to evaluate the stability of the results. Through sensitivity analysis, individual studies were eliminated item by item. After the exclusion of each literature, and re-integration of effect values, all were within the CI, and there was no statistical difference compared with that before removal (*I*^2^ = 0), indicating that the sensitivity of the included literature was low, and that the results of this study are stable.

## 4 Discussion

MicroRNAs are potentially related to the prognosis of heart failure patients. The prognosis of heart failure remains challenging, especially when patients show no significant signs of capacity overload. In such a context, the objectivity, repeatability and reliability of MicroRNA as a biomarker would complement the gaps in research. In addition, potential miRNA biomarkers could guidance of short and long-term therapy in acute heart failure (AHF) patient. Accurate outcome prediction can be used clinically to select the best of several available therapies for AHF patient. Currently, the only recommended biomarkers in this regard are NT-BNP, NT-proBNP, and highly-sensitive C-reactive protein (hs-CRP), whose elevated levels can predict the prognosis of HF patients to some extent, but still have great limitations and cannot accurately determine patient's prognosis.

There are 3 repetitive miRNAs (*miR-30*, *miR-423-5p*, and *miR-18*) among all relevant microRNAs found in all 4 studies. All these 3 miRNAs are associated with worse overall survival of heart failure patients: *miR-423-5p* (HR = 0.66, 95% CI = 0.53-0.84), *miR-30* (HR = 0.63, 95% CI = 0.46-0.85), and *miR-18* (HR = 0.59, 95% CI = 0.43-0.80). For the repetitive miRNAs, different studies find different members of the same family (e.g., *miR-18a-5p* and *miR-18b-5p*; *miR-30d* and *miR-30e-5p*). However, *miR-423-5p* is the same for different studies. *miR-423-5p* is repeatedly used in several studies.^[29]^ The low levels of miR-423-5p in plasma are associated with poor prognosis in HF patients, registering a higher mortality rate.^[32]^ MicroRNAs are expected to greatly improve the accuracy of predicting the outcome of patients with heart failure.

## 5 Limitations

Some limitations in this study include: Many variables can affect the results of meta-analysis, such as different types of samples (race, plasma, serum, and tissue), disease stage, miRNA method and disconnection value^[36]^; This analysis only contains English literature, excluding articles of other languages; This study excludes papers that reported survival curves without Hazard Rate (HR) or 95% CI, which reduces the number of included papers^[37–39]^; Due to the update of the heart failure guidelines, there are differences in the data extraction of heart failure in some literature. We kept the data to try to have a complete summary of the characteristics of the data as much as possible. The inclusion criteria of this work may have excluded relevant miRNA literature of prognostic value. For future clinical and scientific research, this study is the first to systematically assess the association between the expression of circulating miRNAs and disease development in heart failure patients. Future clinical and scientific research may benefit from the findings of this study.

## 6 Conclusions

In summary, circulating *miR-423-5p, miR-126, miR-23, miR-21, miR-1, miR-30d, miR-18a-5p, miR-18b-5p, miR-16-5p, miR-27a-3p, miR-26b-5p, miR-30e-5p, miR-423-3p, miR-301a-3p, miR-233-3p, miR-199a-3p, miR-128, miR-106a-5p*, and *miR-652-3p* demonstrate significant prognostic value. Among them, *miR-423-5p, miR-18a-5p, miR-18b-5p, miR-30d*, and *miR-30e-5p* are strong biomarkers of prognosis in HF.

## 7 Summary and outlook

In recent years, miRNAs and their potential association between microRNA and the onset of heart failure have attracted more and more attention.^[40–43]^ Increasing numbers of studies are addressing the uncertainties on the origins, transport function and mechanisms of action of miRNAs providing important information for future clinical applications. Several miRNAs have been implicated in factors of development and progression of heart failure, such as cardiac hypertrophy, fibrosis, and apoptosis. Studies in miRNAs increase the understanding of heart failure at a deeper pathophysiology level and may provide new means for clinical diagnosis, prognosis and even treatment of heart failure.

## Acknowledgments

We thank Edit Ideas for its linguistic assistance.

## Author contributions

**Conceptualization:** Jie Yang, Chuan-Hua Yang.

**Data curation:** Jie Yang, Xue-Song Yang, Chuan-Hua Yang.

**Formal analysis:** Jie Yang, Xue-Song Yang.

**Funding acquisition:** Jie Yang.

**Investigation:** Shao-Wei Fan, Xiao-Yu Zhao, Chao Li, Zheng-Yao Zhao, Hui-Juan Pei, Lu Qiu.

**Methodology:** Jie Yang, Xue-Song Yang, Chuan-Hua Yang.

**Project administration:** Jie Yang, Xue-Song Yang.

**Resources:** Jie Yang.

**Software:** Jie Yang.

**Supervision:** Jie Yang, Xue-Song Yang, Chuan-Hua Yang.

**Validation:** Jie Yang.

**Visualization:** Jie Yang.

**Writing – original draft:** Jie Yang, Xue-Song Yang, Shao-Wei Fan, Xiao-Yu Zhao, Chao Li, Zheng-Yao Zhao, Hui-Juan Pei, Lu Qiu, Xin Zhuang.

**Writing – review & editing:** Xin Zhuang, Chuan-Hua Yang, Jie Yang.
